# Priming nonlinear searches for pathway identification

**DOI:** 10.1186/1742-4682-1-8

**Published:** 2004-09-14

**Authors:** Siren R Veflingstad, Jonas Almeida, Eberhard O Voit

**Affiliations:** 1 Department of Chemistry, Biotechnology and Food Science, Agricultural University of Norway, N-1432 Ås, Norway; 2 Center for Integrative Genetics (Cigene), Agricultural University of Norway, N-1432 Ås, Norway; 3 Department of Biostatistics, Bioinformatics and Epidemiology, Medical University of South Carolina, 303K Cannon Place, 135 Cannon Street, Charleston, SC 29425, USA; 4 Department of Biochemistry and Molecular Biology, Medical University of South Carolina, 303K Cannon Place, 171 Ashley Avenue, Charleston, SC 29425, USA

## Abstract

**Background:**

Dense time series of metabolite concentrations or of the expression patterns of proteins may be available in the near future as a result of the rapid development of novel, high-throughput experimental techniques. Such time series implicitly contain valuable information about the connectivity and regulatory structure of the underlying metabolic or proteomic networks. The extraction of this information is a challenging task because it usually requires nonlinear estimation methods that involve iterative search algorithms. Priming these algorithms with high-quality initial guesses can greatly accelerate the search process. In this article, we propose to obtain such guesses by preprocessing the temporal profile data and fitting them preliminarily by multivariate linear regression.

**Results:**

The results of a small-scale analysis indicate that the regression coefficients reflect the connectivity of the network quite well. Using the mathematical modeling framework of Biochemical Systems Theory (BST), we also show that the regression coefficients may be translated into constraints on the parameter values of the nonlinear BST model, thereby reducing the parameter search space considerably.

**Conclusion:**

The proposed method provides a good approach for obtaining a preliminary network structure from dense time series. This will be more valuable as the systems become larger, because preprocessing and effective priming can significantly limit the search space of parameters defining the network connectivity, thereby facilitating the nonlinear estimation task.

## Introduction

The rapid development of experimental tools like nuclear magnetic resonance (NMR), mass spectrometry (MS), tissue array analysis, phosphorylation of protein kinases, and fluorescence labeling combined with autoradiography on two-dimensional gels promises unprecedented, powerful strategies for the identification of the structure of metabolic and proteomic networks. What is common to these techniques is that they allow simultaneous measurements of multiple metabolites or proteins. At present, these types of measurements are in their infancy and typically limited to snapshots of many metabolites at one time point (*e.g.*, with MS; [1,2]), to short time series covering a modest number of metabolites or proteins (*e.g.*, with NMR [3,4], 2-d gels [5] or protein kinase phosphorylation [6]), or to tissue arrays [7] that permit the simultaneous high-throughput analysis of proteins in a single tissue section by means of antibody binding or MS. Nonetheless, it is merely a matter of time that these methods will be extended to relatively dense time series of many concentration or protein expression values. We will refer to these types of data as metabolic or proteomic *profiles *and to the time development of a single variable within such a composite profile as *trace*. The intriguing aspect of profiles is that they implicitly contain information about the dynamics and regulation of the pathway or network from which the data were obtained. The challenge for the mathematical modeler is thus to develop methods that extract this information and lead to insights about the underlying pathway or network.

In simple cases, the extraction of information can be accomplished to some degree by direct observation and interpretation of the shape of profiles. For instance, assuming a pulse perturbation from a stable steady state, Vance *et al. *[8] present guidelines for how relationships between the perturbed variable and the remaining variables may be deduced from characteristics of the resulting time profiles. These characteristics include the direction and timing of extreme values (*i.e.*, the maximum deviation from steady state) as well as the slopes of the traces at the initial phase of the response. Torralba *et al. *[9] recently demonstrated that these guidelines, applied to a relatively small set of experiments, were sufficient to identify the first steps of an *in vitro *glycolytic system. Similarly, by studying a large number of perturbations, Samoilov *et al. *[10] showed that it is possible to quantify time-lagged correlations between species and to use these to draw conclusions about the underlying network.

For larger and more complex systems, simple inspection of peaks and initial slopes is not feasible. Instead, the extraction of information from profiles requires two components. One is of a mathematical nature and consists of the need for a model structure that is believed to have the capability of capturing the dynamics of the underlying network structure with sufficient accuracy. The second is computational and consists of fitting this model to the observed data. Given these two components along with profile data, the inference of a network is in principle a regression problem, where the aim is minimization of the distance between the model and the data. If a linear model is deemed appropriate for the given data, this process is indeed trivial, because it simply requires multivariate linear regression, which is straightforward even in high-dimensional cases. However, linear models are seldom valid as representations of biological data, and the alternative of a nonlinear model poses several taxing challenges.

First, in contrast to linear models, there are infinite possibilities for nonlinear model structures. In specific cases, the subject area from which the data were obtained may suggest particular models, such as a logistic function for bacterial growth, but in a generic sense there are hardly any guidelines that would help with model selection. One strategy for tackling this problem is the use of *canonical forms*, which are nonlinear structures that conceptually resemble the unalterable linear systems models, but are nonlinear. Canonical models have in common that they always have the same mathematical structure, no matter what the application area is. They also have a number of desirable features, which include the ability to capture a wide variety of behaviors, minimal requirements for *a priori *information, clearly defined relationships between network characteristics and parameters, and greatly enhanced facility for customized analysis.

The best-known examples of nonlinear canonical forms are Lotka-Volterra models (LV; [11]), their generalizations [12], and power-law representations within the modeling framework of Biochemical Systems Theory (BST; [13-15]), most notably Generalized Mass Action (GMA) systems and S-systems. Lotka-Volterra models have their origin in ecology and focus strictly on interactions between two species at a time. Well-studied examples include competition processes between species, the dynamics of predators and prey, and the spread of endemic infections. In the present context it might seem reasonable to explore the feasibility of these models for the representation of the dynamics of proteins and transcription factor networks, but this has not been done so far.

The strict focus on two-component interactions in LV models has substantial mathematical advantages, but it has proven less convenient for the representation of metabolic pathways, where individual reaction steps depend on the substrate, but not necessarily on the product of the reaction, or are affected by more than two variables. A simple example of the latter is a bi-substrate reaction that also depends on enzyme activity, a co-factor and possibly on inhibition or modulation by some other metabolite in the system. These types of processes have been modeled very successfully with GMA and S-systems. Between these two forms, the S-system representation has unique advantages for system identification from profiles, as was shown elsewhere [16-24] and will be discussed later in this article. In some sense, Karnaukhov and Karnaukhova [25] used a very simplified GMA system for biochemical system identification from dynamic data, in which all mono-substrate or bi-substrate reactions were of first order. This reduced the estimation to the optimization of rate constants, which the authors executed with an integral approach.

The inference of a nonlinear model structure from experimental data is in principle a straightforward "inverse problem" that should be solvable with a regression method that minimizes the residual error between model and data. In practice, however, this process is everything but trivial (*cf. *[26]) as it almost always requires an iterative search algorithm with all its numerical challenges, such as the existence of multiple local minima and failure to converge. Recent attempts of ameliorating this problem have included Bayesian inference methods [27], similarity measures and correlation [28], mutual information [29], and genetic algorithms [30]. An indication of the complexity of nonlinear estimation tasks and their solutions is a recent pathway identification involving an S-system with five variables, which was based on a genetic algorithm [21]. The algorithm successfully estimated the parameter values, but although the system under study was relatively small and noise free, each loop in the algorithm took 10 hours on a cluster of 1,040 Pentium III processors (933 MHz). It is quite obvious that such an approach cannot be scaled up to systems of dozens or hundreds of variables.

Nonlinear estimation methods have been studied for a long time, and while computational and algorithmic efficiency will continue to increase, the combinatorial explosion of the number of parameters in systems with increasingly more variables mandates that identification tasks be made easier if larger systems are to be identified. One important possibility, which we pursue here, is to prime the iterative search with high-quality starting conditions that are better than naïve defaults. Clearly, if it is possible to identify parameter guesses that are relatively close to the true, yet unknown solution, the algorithm is less likely to get trapped in suboptimal local minima. We are proposing here to obtain such initial guesses by preprocessing the temporal profile data and fitting them preliminarily by straightforward multivariate linear regression. The underlying assumption is that the structural and regulatory connectivity of the network will be reflected, at least qualitatively, in the regression coefficients. D'haeseleer *et al. *[31] explored a similar approach for analyzing mRNA expression profiles, but could not validate their results because they lacked a mechanistic model of gene expression. Furthermore, because of the unique relationship between network structure and parameters in S-system models (see below), we will demonstrate that it is possible to translate the regression coefficients into constraints on the parameter values of an S-system model and thereby to reduce the parameter search space very dramatically.

Several other groups have recently begun to target network identification tasks with rather diverse strategies. Chevalier *et al. *[32] and Diaz-Sierra and co-workers [33,34] proposed an identification approach that is similar to the one proposed here in some aspects, though not in others. These authors also used linearization of a nonlinear model, but based their estimation on measured time developments of the system immediately in response to a small perturbation. These measurements were used to estimate the Jacobian of the system at the steady state. In contrast to this focus on a single point, we are here using smoothed long-term time profiles and do not necessarily require system operation at a steady state. Also using linearization, Gardner *et al. *[35] recently proposed a method of network identification by multiple regression. However, they only considered steady-state measurements as opposed to temporal profiles. It is known from theoretical analyses (*e.g.*, [15,36]) that different dynamical models may have the same steady state and that therefore steady-state information alone is not sufficient for the full characterization of a network. Mendes and Kell [37] used a neural network approach for an inverse problem in metabolic analysis, but their target system was very small and fully known in structure. Furthermore, their data consisted of a "large number of steady-state simulations", rather than the limited number of time traces on which our analysis is based. Chen *et al. *[38] used neural networks and cubic splines for smoothing data and identifying rate functions in otherwise linear mass-balance models.

## Methods

The behavior of a biochemical network with *n *species can often be represented by a system of nonlinear differential equations of the generic form



where **X **is a vector of variables *X*_*i*_, *i *= 1, ..., *n*, **f **is a vector of nonlinear functions *f*_*i*_, and *μ *is a set of parameters. If the mathematical structure of the functions *f*_*i *_is known, the identification of the network consists of the numerical estimation of *μ*. In addition to the challenges associated with nonlinear searches mentioned above, this estimation requires numerical integration of the differential equations in (1) at every step of the search. This is a costly process, requiring in excess of 95% of the total search time; if the differential equations are stiff, this percentage approaches 100% [39]. A simplification, which circumvents the problem of integration, consists of substituting the system of differential equations with decoupled algebraic equations by replacing the differentials on the left-hand side of Eq. (1) with estimated slopes [16,17]. Thus, if the system consists of *n *differential equations, and if measurements are available at *N *time points, the decoupling leads to *n *× *N *algebraic equations of the form



It may be surprising at first that it is valid to decouple the tightly coupled system of nonlinear differential equations. Indeed, this is only justified for the purpose of parameter estimation, where the decoupled algebraic equations simply provide numerical values of variables (metabolites or proteins) and slopes at a finite set of discrete time points. The experimental measurements thus serve as the "data points," while the parameters *μ*_*ij *_are the "unknowns" that need to be identified.

The quality of this decoupling approach is largely dependent on an efficient and accurate estimation of slopes from the data. Since the data must be expected to contain noise, this estimation is *a priori *not trivial. However, we have recently shown [23,39] that excellent estimates can be obtained by smoothing the data with an artificial neural network and computing the slopes from the smoothed traces (see Appendix for detail).

### Different Linearization Approaches

The smoothing and decoupling approach reduces the cost of finding a numerical solution of the estimation task considerably. Nonetheless, algorithmic issues associated with local minima and the lack of convergence persist and can only be ameliorated with good initial guesses. To this end, we linearize the model **f **in Eq. (1) about one or several reference states. As long as the system stays close to the given reference state(s), this linearization is a suitable and valid approximation. We consider four options: (I) linearization of absolute deviations from steady state; (II) linearization of relative deviations from steady state; (III) piecewise linearization; and (IV) Lotka-Volterra linearization.

Option (I) is based on deviations of the type *z*_*i *_= *X*_*i *_- *X*_*ir*_, where *X*_*ir *_denotes the value at a reference state of choice. If the reference state is chosen at a stable steady state, the first-order Taylor-approximation is given by



where **A **is the *n × n *Jacobian with elements *a*_*ij *_= (*df*_*i *_/ *dX*_*j*_) calculated at **X**_*r *_(*cf. *[32-34]). If the reference state is not chosen at a steady state, the equation contains an additional constant term *a*_*i*0_, which is equal to *f*_*i*_(**X**_*r*_).

For option II, we define a new variable *u*_*i *_= *z*_*i*_/*X*_*ir*_. At a steady state, this yields the linear system



where **A' **is an *n × n *matrix in which *a'*_*ij *_= (*X*_*jr *_/ *X*_*ir*_)·*a*_*ij*_.

A general concern regarding linearization procedures is the range of validity of sufficiently accurate representation, which is impossible to define generically. From an experimental point of view, the perturbations from steady state must be large enough to yield measurable responses. This may require that they be at the order of 10% or more. Depending on the nonlinearities in **f**, a perturbation of this magnitude may already lead to appreciable approximation errors. While this is a valid argument, it must be kept in mind that the purpose of this priming step is simply to detect the topological structure of connectivity and not necessarily to estimate precise values of interaction parameters. Simulations (see below) seem to indicate that this detection is indeed feasible in many cases, even if the deviations are relatively large.

In order to overcome the limitation of small perturbations, a piecewise linear regression (option III) may be a superior alternative. In this case, we subdivide the dataset into appropriate time intervals and linearize the system around a chosen state within each subset. Most (or all) reference states are now different from the steady state, with the consequence that Eq. (3) has a constant term *a*_*i*0_, which is equal to *f*_*i*_(**X**_*r*_). The choice of subsets and operating points offers further options. In the analysis below, we use the locations of extreme values (maximum deviation from steady state) of the variables as the breakpoints between different subsets. Thus, a variable with a maximum and a later minimum has its time course divided into three subsets.

The fourth alternative (option IV) is a Lotka-Volterra linearization. In a Lotka-Volterra model, the interaction between two species *X*_*i *_and *X*_*j *_is assumed to be proportional to the product *X*_*i*_*X*_*j *_[11]. Furthermore accounting for linear dependence on the variable of interest itself, the typical Lotka-Volterra equation for the rate of change in *X*_*i *_is



The right-hand side of this nonlinear differential equation becomes linear if both sides are divided by *X*_*i*_, which is usually valid in biochemical and proteomic systems, because all quantities of interest are non-zero. Thus, the differentials are again replaced by estimated slopes, the slopes are divided by the corresponding variable at each time point, and fitting the nonlinear LV model to the time profiles becomes a matter of linear regression that does not even require the choice of a reference state. The quality of this procedure is thus solely dependent on the quality of the data and ability of the LV model to capture the dynamics of the observed network. It is known (*e.g.*, [11,40]) that the mathematical structure of LV models is rich enough to model any nonlinearities, if sufficiently many equations are included. However, there is no general information about the quality of fit in particular modeling situations.

### Regression

No matter which option is chosen, the next step of the analysis consists of subjecting all measured time traces to multivariate linear regression and solving for the regression coefficients (*i.e.*, *v*_*ij*_'s and *w*_*i*_'s, or *α*_*ij*_'s). The response variable is the rate of change of a metabolite, while the predictors are the concentrations of each metabolite in the network. The different linearization models (I-IV) differ in the transformations of the original datasets, which are summarized in Table 1. For example, the response variable of the linear model in Eq. (4) is given by *y*_*i *_=  /*X*_*ir*_, and the predictor variables are transformed as *x*_*i *_= (*X*_*i *_- *X*_*ir*_)/*X*_*ir*_.

**Table 1:** Transformation of data for regression analysis

	*RESPONSE VARIABLE*	*PREDICTOR VARIABLE*
A. Absolute deviation from a reference state	*y*_*i *_=	*x*_*i *_= *X*_*i *_- *X*_*ir*_
B. Relative deviation from a reference state		
C. Lotka-Volterra system		*x*_*i *_= *X*_*i*_

We assume the general linear model is *y *= *a*_*i*0 _+ Σ(*a*_*ij *_x_*j*_). The *X*_*i *_denote experimental time series data for metabolite *i*, while the slopes () are estimated from the smooth output functions of the artificial neural network that had been trained on the experimental data. Subscript *r *denotes the value of the metabolite at a reference state. Linearization options I and II are included in transformations A and B respectively, assuming that the reference state is a steady state. For a piecewise linear linearization (option III), the data may be transformed following either A or B.

The result of the regression is a matrix of coefficients that indicate to what degree a metabolite *X*_*j *_affects the dynamics (slope) of another metabolite *X*_*i*_. In particular, a coefficient that is zero or close to zero signals that there is no significant effect of *X*_*j *_on the slope of *X*_*i*_. By the same token, a coefficient that is significantly different from zero suggests the presence of an effect, and its value tends to reflect the strength and direction of the interaction. In either case, the coefficients computed from the linear regression provide valuable insight into the connectivity of the network. Furthermore, the estimated coefficients provide constraints on the parameter values of the desired nonlinear model **f**. Indeed, if **f **consists of an S-system model, the coefficients estimated from the regression can be converted into combinations of S-system parameters, as is demonstrated in the following theoretical section and illustrated later with a specific example.

### Relationships between Estimated Regression Coefficients and S-system Parameters

The regression analysis yields coefficients that offer information on the connectivity of the network of interest. It also provides clues about the parameter values of the underlying nonlinear network model **f **in Eq. (1) if this model has the form of an S-system. To determine the relationships between the regression coefficients and the parameters of the S-system, it is convenient to work backwards by computing the different types of linearizations discussed before for the particular case of S-system models. This derivation is simply a matter of applying Taylor's theorem.

In the S-system formalism, the rate of change in each pool (variable) is represented as the difference between influx into the pool and efflux out of the pool. Each term is approximated by a product of power-law functions, so that the generic form of any S-system model is



where *n *is the number of state variables [13,14]. The exponents *g*_*ij *_and *h*_*ij *_are called *kinetic orders *and describe the quantitative effect of *X*_*j *_on the production or degradation of *X*_*i*_, respectively. A kinetic order of zero implies that the corresponding variable *X*_*j *_does not have an effect on *X*_*i*_. If the kinetic order is positive, the effect is activating or augmenting, and if it is negative, the effect is inhibiting. The multipliers *α *_*i *_and *β *_*i *_are *rate constants *that quantify the turnover rate of the production or degradation, respectively.

If the Taylor linearization is performed at a steady state, the production term of the S-system model equals the degradation term. The absolute deviation of the first option, *z*_*i *_= *X*_*i *_- *X*_*is*_, where the subscript *s *denotes the value of the variable at steady state, then leads directly to



where

**Figure 1:**
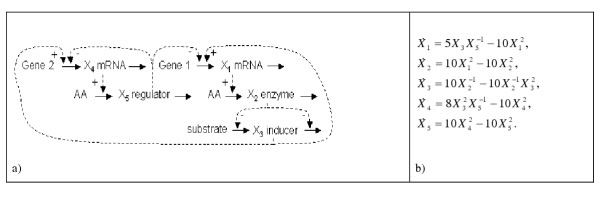
**Test System. **a) Gene network [42] used as test system for illustrating the proposed methods. Solid arrows represent material flow, while dashed arrows indicate regulatory signals that either activate (+) or inhibit (-) a process. The network contains two genes, Gene 1 and 2. *X*_1 _is the mRNA produced from gene 1, *X*_2 _is the enzyme for which the gene codes, and *X*_3 _is an inducer protein catalyzed by *X*_2_. *X*_4 _is the mRNA produced from Gene 2 and *X*_5 _is a regulator protein for which the gene codes. Positive feedback from *X*_3 _and negative feedback from *X*_5 _are assumed in the production of mRNAs from the two genes. b) S-system model of the gene network, according to Hlavacek and Savageau [42] and Kikuchi *et al. *[21].

*c*_*ij *_= *g*_*ij *_- *h*_*ij*_,



(*cf*. [41]). The so-called F-factors *F*_*ij *_are always non-negative, while *c*_*ij *_may be either positive or negative depending on the relationship between *X*_*i *_and *X*_*j*_. A common scenario is that a variable *X*_*j *_influences either the production or degradation of variable *X*_*i*_, but not both. In this case, a positive (negative) *c*_*ij *_implies activation (inhibition) of production or inhibition (activation) of degradation. The special case of *c*_*ij *_= 0 permits two possible interpretations: 1) *g*_*ij *_= *h*_*ij *_= 0, which implies that *X*_*j *_has no effect on either production or degradation of *X*_*i*_; or 2) *g*_*ij *_= *h*_*ij *_≠ 0, which means that *X*_*j *_has the same effect on both production and degradation of *X*_*i*_. The former case is the more likely, but there are examples where the latter may be true as well, and this is indeed the case in the small gene network in Figure 1.

Comparing the expression in Eq. (6) with the linear regression results, one sees immediately that each coefficient *a*_*ij *_in Eq. (3) corresponds to the product of *F*_*ij *_and *c*_*ij*_:

*a*_*ij *_= *F*_*ij*_*c*_*ij*_.     (7)

Thus, once the regression has been performed and the coefficients *a*_*ij *_have been estimated, the parameters of the corresponding S-system are constrained – though not fully determined – by Eq. (7). In particular, Eq. (7) does not allow a distinction between various combinations of *g*_*ij *_and *h*_*ij*_, as long as the two have the same difference. For instance, re-interpreting the regression coefficients as S-system parameters does not differentiate between the overall absence of effect of *X*_*j *_on *X*_*i *_(*g*_*ij *_= *h*_*ij *_= 0) and the same effect of *X*_*j *_on both the production and degradation of *X*_*i *_(*g*_*ij *_= *h*_*ij *_≠ 0). This observation is related to the observation of Sorribas and Cascante [36] that steady-state measurements are insufficient for completely identifying an S-system model.

Relative deviations from steady state, *u*_*i *_= (*X*_*i *_- *X*_*is*_) / *X*_*is*_, in option II, are assessed in an analogous fashion. In this case one obtains



where

*c*_*ij *_= *g*_*ij *_- *h*_*ij*_,



[41]. Again, the F-factors *F*_*i *_are positive, while *c*_*ij *_may be either positive or negative.

The piecewise linear model for an S-system is easily derived as well. It is given as



where *X*_*jr *_denotes the value of the variable at the reference state. This case also includes the situation of a single approximation, which however is not necessarily based on a steady-state operating point.

In the case of the Lotka-Volterra linearization, the correspondence between computed regression coefficients and S-system parameters is determined most easily by dividing the S-system equations by the corresponding *X*_*i *_and then linearizing around an operating point. The resulting expressions become especially simple if this point is chosen as the steady state. In this case, the relationship between the parameters of the LV system and the S-system are



where *c*_*ij *_= *g*_*ij *_- *h*_*ij*_.

## Results

We applied the methods described in the previous sections to simulated time profiles obtained from the small gene network in Figure 1a. Hlavacek and Savageau [42] modeled this network as an S-system with five differential equations (Figure 1b), and Kikuchi *et al. *[21] used it recently for exploring computational features of their proposed structure identification algorithm. The benefit of working with a known model is that complete information is available about both its structure and parameter values. In particular, it is possible to perform any number of experiments and to produce data and slopes with predetermined noise levels, which is not typically possible with real data. For this analysis, we thus used simulated noise free "data," which allowed us to skip the neural network step of smoothing [23,39].

To generate time profiles, the system was implemented with the parameter values published by Hlavacek and Savageau [42], and as in the analysis of Kikuchi *et al. *[21], the model was initialized with various perturbations from steady state and numerically integrated over a sufficient time horizon to allow the system to return to the steady state.

### Preliminary Analysis

**Figure 2:**
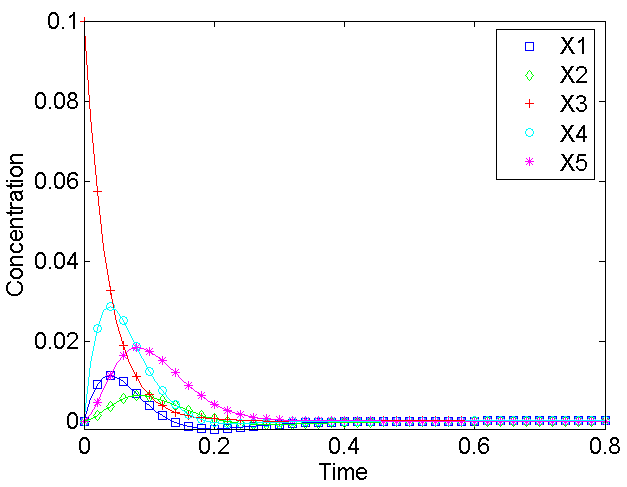
**Dynamic response of the network after a perturbation in *X*_3 _**The response is shown as relative deviation from steady state. The guidelines proposed by Vance *et al. *[8] indicate that *X*_1 _and *X*_4 _precede *X*_2 _and *X*_5 _because they reach their maximum deviation earlier and the maximal values are larger than those of *X*_2 _and *X*_5_. All variables respond in a positive manner, which implies either a mass transfer or positive modulation (activation). The system determined from this analysis is essentially the same as in Figure 1a. The only relationship missed is the effect of *X*_2 _on the production and degradation of *X*_3_.

Quasi as a pre-analysis, we examined the guidelines proposed by Vance *et al. *[8]. Indeed, the results show that many of these are applicable to the gene regulatory network. The order of the extrema (*i.e.*, the maximum deviations from steady state) of the various variables both in time and size is in accordance with their "topological distance" from the perturbed variable, and variables not directly affected by the perturbed variable have zero initial slopes. As an example, the effect of a perturbation in *X*_3 _is shown in Figure 2. All variables increase in response, with variables *X*_1 _and *X*_4 _reaching their maximal deviation from steady state before *X*_2 _and *X*_5_, suggesting that *X*_1 _and *X*_4 _precede *X*_2 _and *X*_5 _in the pathway. The value of the initial slope is different from zero for *X*_1 _and *X*_4_, implying that these variables are directly affected by *X*_3_, whereas *X*_2 _and *X*_5 _have zero initial slopes suggesting that their responses are mediated through other variables.

Maximal information about the network is obtained when every variable is perturbed sequentially. Experimentally, such perturbations could be implemented with modern methods of RNA interference [43] or, for biotechnological purposes, in a chemostat [9]. In our model case, we can actually identify all kinetic orders that are zero in the original model, and this amounts to determining the connectivity of the pathway. The only relationship this analysis does not pick up is the effect of *X*_2 _on *X*_3_. This result is not surprising, because the effect of *X*_2 _is the same on both the production and degradation of *X*_3_, which leads to cancellation. It is noted that this analysis does not necessarily distinguish between transfer of mass and a positive modulation, because both result in a positive effect on a variable. In a realistic situation, biological knowledge may exclude one of the two options, as in this case, where modulation is the only possibility for the effect of *X*_3 _on both *X*_1 _and *X*_4_, because the former is a protein and the latter are RNA transcripts. For the mathematical model in the S-system form, this is not an issue, as both types of influence are included in the equations in the same way (as a positive kinetic order).

### Regression Analysis

While Vance's method works well in this simple noise-free system, it is not scalable to larger and more complex systems. The next step of our analysis is therefore regression according to the four options presented above and with a number of simulated datasets of the gene network that differ in the variable to be perturbed and the size of the perturbation. Because the illustration here uses a known model and artificial data, it is easy to compute the true regression coefficients through differentiation of the S-system model. These coefficients can be used as a reference for comparisons with coefficients computed from the entire time traces, which mimics the estimation process for (smoothed) actual data.

#### Options I, II and IV

The results for three of the options (I, II and IV) can be summarized in the following three points, while the piecewise linear model will be discussed afterwards.

(1) *The network connectivity is reflected in the values of the regression coefficients. *The values of the estimated coefficients provide strong indication as to which variables have a significant influence on the dynamics of other variables. A comparison between computed and estimated coefficients is shown in Table 2 for the linear model with relative deviations (option II, Eq. 8). Most of the coefficients that in reality are zero (for example *a*_12 _and *a*_24_) are not estimated as exactly zero, but their values are at least one order of magnitude smaller than the coefficients that are in actuality not zero. Table 2 also indicates that not all coefficients reflect the network correctly. The linear regression gives especially poor estimates for the coefficients associated with variables *X*_3 _and *X*_4_. A possible explanation for *X*_3 _is that the effect of *X*_2 _is present in the non-linear system, but not in the linear system, and thus the behavior of *X*_3 _must be explained by the other variables. Overall, of the 25 theoretically possible connections, 76% are correctly identified, while 24 % are false positives.

**Table 2:** Comparison of computed and estimated coefficients

	**Computed coefficients**	**Estimated coefficients**
**a10**	0	0.0000
**a11**	-14.6780	-14.3647
**a12**	0	-0.1466
**a13**	7.3390	7.3414
**a14**	0	-0.2165
**a15**	-7.3390	-7.1723
**a20**	0	0.0000
**a21**	14.6780	14.6119
**a22**	-14.6780	-14.6540
**a23**	0	-0.0009
**a24**	0	0.0494
**a25**	0	-0.0309
**a30**	0	0.0000
**a31**	0	-2.3527
**a32**	0	1.3989
**a33**	-27.2517	-27.9204
**a34**	0	1.7491
**a35**	0	-0.9955
**a40**	0	0.0000
**a41**	0	2.0843
**a42**	0	-1.0925
**a43**	18.5664	19.0295
**a44**	-18.5664	-20.2112
**a45**	-9.2832	-8.3594
**a50**	0	0.0000
**a51**	0	-0.4026
**a52**	0	0.1384
**a53**	0	-0.0059
**a54**	18.5664	18.8987
**a55**	-18.5664	-18.7852

Regression coefficients for the small gene network (Figure 1), linearized about the steady state and based on relative deviations (option II). The first and second columns contain the computed and estimated regression coefficients, respectively. The regression coefficients *a*_*ij *_refer to the influence of variable *j *on variable *i*, while *a*_*i*0 _is the constant term in each regression model. As the table indicates, the correspondence is good, except for the coefficients relating to *X*_3 _and *X*_4 _(see Text for explanation). The dataset consisted of 401 data points in the interval [0,4] and resulted from a simulation in which *X*_3 _was perturbed at *t *= 0 to a value 5% above its steady-state value.

(2) *The different linear models give (qualitatively) the same results. *A comparison of the results of the three models reveals that the values of the regression coefficients are very similar (see Table 3). The same applies to their signs. Most important, all models correctly identify the connections present in the gene network. They also equally infer the same incorrect relationships. As an example, consider the coefficients associated with *X*_4_: all models infer the net positive effect of *X*_3 _and the net negative effect of both *X*_4 _and *X*_5_. At the same time, they also suggest that *X*_1 _and *X*_2 _have a significant effect on the dynamics of *X*_4_. In reality, they do not directly influence *X*_4 _(see Figure 1), and it may be that their indirect effect, which is mediated by *X*_3_, is causing the false positive result.

**Table 3:** Comparison of the different linearization options (I, II and IV)

	**I. Absolute deviation**	**II. Relative deviation**	**IV. Lotka-Volterra**
**a10**	0.0000	0.0000	14.4748
**a11**	-14.3647	-14.3647	-18.9581
**a12**	-0.1466	-0.1466	-0.6836
**a13**	5.3878	7.3414	7.3367
**a14**	-0.1712	-0.2165	-0.4694
**a15**	-5.6702	-7.1723	-7.4981
**a20**	0.0000	0.0000	0.0144
**a21**	14.6119	14.6119	19.8910
**a22**	-14.6540	-14.6540	-19.9277
**a23**	-0.0006	-0.0009	-0.0001
**a24**	0.0390	0.0494	0.0472
**a25**	-0.0245	-0.0309	-0.0335
**a30**	0.0000	0.0000	26.4020
**a31**	-3.2058	-2.3527	2.8725
**a32**	1.9062	1.3989	-1.7989
**a33**	-27.9204	-27.9204	-26.6164
**a34**	1.8842	1.7491	-1.5871
**a35**	-1.0724	-0.9955	0.9692
**a40**	0.0000	0.0000	8.0270
**a41**	2.6365	2.0843	6.3364
**a42**	-1.3820	-1.0925	-4.1579
**a43**	17.6654	19.0295	19.0005
**a44**	-20.2112	-20.2112	-23.1319
**a45**	-8.3594	-8.3594	-7.7047
**a50**	0.0000	0.0000	0.0869
**a51**	-0.5092	-0.4026	-0.6617
**a52**	0.1751	0.1384	0.4441
**a53**	-0.0055	-0.0059	-0.0003
**a54**	18.8987	18.8987	20.2939
**a55**	-18.7852	-18.7852	-20.2152

Estimated coefficients for three of the linearization approaches: absolute deviation from steady state (left column), relative deviation from steady state (center column) and Lotka-Volterra linearization (right column). The dataset consisted of 401 data points in the interval [0,4] and resulted from a simulation in which *X*_3 _was perturbed at *t *= 0 to a value 5% above its steady-state value.

(3) *The greater the perturbation, the less accurate is the estimation of the regression coefficients. *The deviation between the estimated and computed coefficients increases as the size of the perturbation increases (see Table 4). For the models obtained by linearizing about the steady state (Eqs. (6) and (8)), this is an expected result, as the Taylor-expansion only gives a valid approximation close to steady state. For these systems, "close" may correspond to a perturbation of less than 5–10% with respect to the steady-state value. Nonetheless, the greater perturbations still give a relatively good picture in terms of the connectivity of the system. For a 5% perturbation, the fraction of correctly identified connections is 76% and for a two-fold perturbation it is still 64 %. Perturbations of more than 5–10 % of the steady state also cause problems for the Lotka-Volterra model, from which one might have expected a higher tolerance as the linearization is independent of a reference state. It seems that the dynamics of the true system in our particular example is about equally well modeled by the nonlinear LV-model as by the linear models.

**Table 4:** The effect of the size of the perturbation

	**Computed**	**5 %**	**10 %**	**50 %**	**200 %**
**a10**	0	0.0000	0.0000	0.0001	0.0008
**a11**	-14.6780	-14.3647	-14.1817	-13.1496	-11.3439
**a12**	0	-0.1466	-0.1429	-0.0671	0.5735
**a13**	7.3390	7.3414	7.3438	7.3598	7.3735
**a14**	0	-0.2165	-0.3673	-1.2462	-2.7619
**a15**	-7.3390	-7.1723	-7.0780	-6.4846	-5.2501
**a20**	0	0.0000	0.0000	0.0000	-0.0003
**a21**	14.6780	14.6119	14.5748	14.4207	14.5029
**a22**	-14.6780	-14.6540	-14.6623	-14.7503	-15.1862
**a23**	0	-0.0009	-0.0016	-0.0054	-0.0070
**a24**	0	0.0494	0.0839	0.2494	0.3462
**a25**	0	-0.0309	-0.0464	-0.1119	-0.0951
**a30**	0	0.0000	0.0000	0.0004	0.0038
**a31**	0	-2.3527	-4.5412	-18.2307	-46.8953
**a32**	0	1.3989	2.6336	9.8422	24.4004
**a33**	-27.2517	-27.9204	-28.5955	-34.0204	-54.4047
**a34**	0	1.7491	3.4009	14.0961	39.3252
**a35**	0	-0.9955	-1.8949	-7.0627	-15.4759
**a40**	0	0.0000	0.0000	-0.0001	0.0001
**a41**	0	2.0843	3.7814	14.7316	41.5863
**a42**	0	-1.0925	-1.7693	-5.5766	-13.2688
**a43**	18.5664	19.0295	19.4964	23.2397	37.1866
**a44**	-18.5664	-20.2112	-21.6608	-31.4631	-58.1065
**a45**	-9.2832	-8.3594	-7.6404	-3.2226	6.5808
**a50**	0	0.0000	0.0000	-0.0001	-0.0015
**a51**	0	-0.4026	-0.6581	-2.5848	-10.1097
**a52**	0	0.1384	0.0830	-0.1317	0.1582
**a53**	0	-0.0059	-0.0110	-0.0435	-0.0879
**a54**	18.5664	18.8987	19.1602	21.0620	27.2722
**a55**	-18.5664	-18.7852	-18.9201	-20.0013	-24.0836

Overall, the estimated coefficients deviate more strongly from the corresponding computed values as the perturbation increases. However, there are substantial differences between variables. The coefficients associated with variable *X*_2_, for example, are hardly influenced, while the coefficients associated with *X*_3 _are strongly affected. Overall, the method seems to produce the best results for perturbation up to 10%. The datasets for the regression consisted of 401 data points in the interval [0,4] and the method of linearization was option II.

#### Option III

The piecewise linear model was obtained by dividing the whole dataset into three smaller subsets for each variable. The first interval contained the data points from *t *= 0 to the time of the first extreme value for a given variable (in this case a maximum for all variables). For the perturbed variable (having its first extreme value at *t *= 0) the first limit point was given by the smallest of the limit points of the other variables. The second interval contained the data points from the first to the second extreme value (a minimum), while the third interval included the remaining data points. The midpoint of each interval was taken to be the reference state. The result of the piecewise linear regression for a 5% deviation in *X*_3 _is given in Table 5. The first subset does not reflect the interactions of the system especially well, whereas the other two subsets correctly classify 88% and 96%, respectively, of the true connections in the network. It is worth noting that the coefficients associated with *X*_3 _in the two last subsets reflect the variable's connectivity to a much greater extent than the other linearization approaches. As the reference state is different from the steady state, the effect of *X*_2 _is present in the linear system as well, and thus there is no compensation through the other variables. Another benefit is that the piecewise model tolerates larger perturbations. Even for a two-fold perturbation, the fraction of correctly identified coefficients in the last subset is 84%.

**Table 5:** Results for piecewise linear regression

	**Interval 1**	**Interval 2**	**Interval 3**
**a10**	0.1315	-0.0419	0.0000
**a11**	-42.3980	-14.1738	-14.5490
**a12**	0.0000	-0.8010	-0.0464
**a13**	8.9105	7.3653	7.6299
**a14**	12.7757	-0.3340	-0.1386
**a15**	-3.3476	-6.9121	-7.2940
**a20**	0.0567	-0.0197	0.0000
**a21**	-1.1939	14.4913	14.6792
**a22**	-32.3300	-14.5116	-14.6784
**a23**	0.6133	0.0057	-0.0205
**a24**	7.0917	0.1016	-0.0018
**a25**	7.9313	-0.1047	0.0067
**a30**	-0.7858	-0.0181	0.0000
**a31**	-130.3724	-0.2358	0.0021
**a32**	0.0000	0.3616	-0.0007
**a33**	-20.7724	-27.6129	-27.2551
**a34**	62.1525	0.3496	-0.0027
**a35**	19.1470	-0.1984	0.0006
**a40**	0.3164	-0.0709	0.0000
**a41**	-13.6819	1.1412	-0.0115
**a42**	0.0000	-2.1478	0.0015
**a43**	19.8295	18.8534	18.6927
**a44**	-13.3654	-19.5811	-18.5494
**a45**	-7.2135	-8.0985	-9.2792
**a50**	0.1617	-0.0393	0.0000
**a51**	-149.5199	-0.8195	0.0250
**a52**	-160.3341	0.8175	-0.0074
**a53**	5.7537	0.0580	-0.0304
**a54**	85.3050	19.0394	18.5356
**a55**	53.9745	-19.1183	-18.5623

The complete dataset is divided into three subsets for each variable, where the first and second extreme values serve as breakpoints. The datasets for the regression consisted of 401 data points in the interval [0,4] and resulted from a simulation in which *X*_3 _was perturbed at *t *= 0 to a value 5% above its steady-state value.

#### Degree of Similarity as a Measure of Reliability

If we compare the results of all four linearized models, the degree of similarity may provide a measure of how reliable the estimated coefficients are, assuming that an interaction identified in all models is more reliable than an interaction identified in only one or few of the models. Considering the piecewise linear model as three models, yielding a total of 6 models from one dataset, one may thus determine the most likely connectivity for the small gene network. The result is presented in Table 6. Of the 25 possible connections, 12 were identified correctly in all models, either as being positive, negative or non-existent, while an additional 6 connections were correctly identified in either 4 or 5 of the six models. For these six, one of the models misidentifying the type of connection was the first subset of the piecewise linear approximation, which does not reflect the connectivity of the network especially well, as was shown in Table 5. It is also worth noting that only one of the interactions associated with *X*_3 _is identified correctly from comparing the six models. The classification of the remaining four connections varies greatly among the different models, and it is therefore impossible to deduce a type of interaction with sufficient reliability.

**Table 6:** Collective inference of the gene network based on results from all linearizations

*X*1	*X*2	*X*3	*X*4	*X*5	
X1	**-** (100 %)	**0** (67 %)	**+** (100 %)	**0** (83 %)	**-** (100 %)
X2	**+** (100 %)	**-** (100 %)	**0** (100 %)	**0** (83 %)	**0** (83 %)
X3	?	?	**-** (100 %)	?	?
X4	+ (67 %)	- (67 %)	**+** (100 %)	**-** (100 %)	**-** (100%)
X5	- (83 %)	**0** (83 %)	**0** (83 %)	**+** (100 %)	**-** (100 %)

Each minus sign implies a negative influence; a plus sign implies a positive influence, while zero implies no influence. Bold symbols denote correctly identified interactions, and numbers in parentheses give the fraction of models that suggested positive identification. Question marks imply that no type of interaction was identified in more than 50% of the models.

#### Constraining the Parameter Values

In addition to reflecting the connectivity, the coefficients provide likely parameter ranges or likely constraints on parameter values of the true model. As an example, consider variable *X*_1_. Table 6 indicates that the variables having a significant effect are *X*_1_, *X*_3 _and *X*_5_. If so, the linear model in Eq. (8) suggests the following:



where  and the regression coefficients (*a*_*ij*_) are taken from the model in Eq. (4). The values of the variables at steady state are known. Because the kinetic orders may be positive or negative and the *c*_*ij *_may result from different combinations of *g*_*ij*_'s and *h*_*ij*_'s, it is not possible to deduce directly which exponent is greater than the other. However, in many cases one may have additional information on the system, which further limits the degrees of freedom (*e.g.*, [23]). In addition, the steady-state equation  must be satisfied and provides yet another constraint.

## Discussion

Identifying the structure of metabolic or proteomic networks from time series is a task that most likely will require large, parallelized computational effort. The search space for the algorithms is typically of high dimension and unknown structure and very often contains numerous local minima. This generic and frequent problem may be ameliorated if the search algorithm is provided with good initial guesses and/or constraints on admissible parameter values. Here, we have shown that linear regression may provide such information directly from the types of data to be expected from future experiments. For illustrative purposes, we used artificial data from a known network, but all methods are directly applicable to actual profile data and scaleable to large systems.

The coefficients estimated from the different regressions reflect the effect of one variable on another surprisingly well and thus provide a simple fashion of prescreening the connectivity of the network. In addition, the estimated coefficients provide constraints on the parameter values, if the alleged nonlinear model has the form of an S-system. To explore the pre-assessment of data as fully as feasible, we studied four linearization strategies: using an absolute deviation from steady state; a relative deviation from steady state; piecewise linearization; and Lotka-Volterra linearization. Interestingly, all models gave qualitatively similar results for the analyzed example, and this degree of similarity may provide a measure of how reliable the identified connections are. Specifically, of the 25 possible connections in the small gene network studied, 19 were identified correctly in at least 83 % of the regression analyses.

A concern of any linearization approach is the validity of the linear approximation. However, as long as the perturbation from steady state remains relatively small, the estimated linear model is likely to be a good fit of the actual nonlinear model, at least qualitatively. This limitation may furthermore be alleviated by fitting the profile data in a piecewise linear fashion. As most reference states in this case are different from the steady state, this strategy has the added benefit that more of the true relationships within the nonlinear model are likely to be preserved. As an alternative, one could explore the performance of the so-called "log-linear" model, which is linear in log-transformed variables [44].

The Lotka-Volterra linearization did not perform as well as expected with regard to large perturbations. This may be a consequence of the particular example, which was originally in S-system form rather than in a form more conducive to the LV structure, which emphasizes interactions between pairs of variables. Since it is easy to perform the LV analysis along with the other regressions discussed here, it may be advisable to execute all four analyses.

The illustrative model used for testing the procedure consisted of a relatively small system with only five variables and relatively few interactions. Nonetheless, one should recall that this very system required substantial identification time in a direct estimation approach [21]. In order to check how scaleable the results of the proposed linearization method are, the method should be tested on larger systems. Some preliminary analyses suggest that the method works well, but that the likelihood of misidentified connections may grow with the size of the system, as one might expect. At the same time, experience with actual biological networks, for instance in ecology and metabolism, suggests that larger systems are often more robust in a sense that they do not deviate as much from the steady state as smaller systems. If this trend holds in general, the linearization becomes a more accurate representation as larger networks are being investigated and the proposed methods will therefore yield more reliable initial indicators of network connectivity. Independent of these issues, the methods proposed here will very likely be more valuable for bigger systems than other methods that are presently available, because without some preprocessing of the data and effectively priming the search, as it is proposed here, the combinatorial explosion will most certainly gain the upper hand eventually.

## Competing interests

None declared.

## Authors' contributions

SRV performed the analysis and prepared the results. JS developed and implemented the neural network for computation of slopes. EOV developed the basic ideas and directed the project.

## Appendix

It was recently shown that good parameter estimates of S-system models from metabolic profiles might be obtained by training an artificial neural network (ANN) directly with the experimental data. The result of this training is a so-called *universal function *which smoothes the data with predetermined precision and also allows the straightforward computation of slopes that can be used for network identification purposes. This appendix briefly outlines the procedure; details can be found in Almeida [45] and Voit and Almeida [24]. The ANN consists of three layers; one input layer, one hidden layer and one output layer. The input layer consists of the measurement time points, the hidden layer has no direct biological interpretation, and the output layer contains the metabolite concentrations or levels of protein expression that the ANN is being trained to represent. The node values of the ANN in the hidden layer are calculated from a linear combination of input values with different weights according to a multivariate logistic equation. Similarly, the values of the output layer are determined from linear combinations of the hidden node values with different weights, again using a multivariate logistic function. It is known that this type of nested multivariate logistic function has unlimited flexibility in modeling nonlinearities [46].

Noise and sample size do not have a devastating effect on the results of the ANN-method, as long as the true trend is well represented [39]. In fact, the ANN approach provides an unlimited number of sampling points, as values at any desired time points may be estimated from the universal output function. Finally, the calculation of the slopes of the smooth output functions is mathematically unwieldy, but computationally straightforward.

The use of the entire time course is in stark contrast to earlier methods of parameter estimation and structure identification in metabolic networks. Mendes and Kell [37] applied their ANN-based parameter estimation to steady-state data, while we are using time profiles.

Chevalier and co-workers [32] first fitted the nonlinear solution with a linear model (as shown in Eq. 3), expressed this solution in terms of eigenvectors and eigenvalues, and then obtained the slopes by numerical differentiation. Sorribas *et al. *[47] suggested a variation on this approach, based on discretizing the solution of Eq. (3) as

*z*(*t*_*k *+ 1_) = *z*(*t*_*k*_)exp(*h*·**A**),     (A1)

where *h *is the step size. The problem is thereby reduced to a mulitilinear regression in which the matrix **Φ **= exp(*h*·**A**) is the output. Instead of estimating the slopes, they obtain the Jacobian directly by  expanded in its Taylor-series. This approach yields a faster convergence to the elements of the Jacobian than the one suggested by Chevalier *et al*. [32], but the regression of Eq. (A1) is very sensitive to noise and missing data points.

Our approach takes advantage of the entire time course and is therefore less sensitive to the particularities of assessing a system at a single point. The ANN itself does not provide much insight, because it is strictly a black-box model, but it is a valuable tool for controlling problems that are germane to any data analysis, namely noise, measurement inaccuracies, and missing data.
